# Nucleotide-Oligomerization-Domain-2 Affects Commensal Gut Microbiota Composition and Intracerebral Immunopathology in Acute *Toxoplasma gondii* Induced Murine Ileitis

**DOI:** 10.1371/journal.pone.0105120

**Published:** 2014-08-20

**Authors:** Markus M. Heimesaat, Ildiko R. Dunay, Marie Alutis, André Fischer, Luisa Möhle, Ulf B. Göbel, Anja A. Kühl, Stefan Bereswill

**Affiliations:** 1 Department of Microbiology and Hygiene, Charité - University Medicine Berlin, Berlin, Germany; 2 Department of Microbiology and Hygiene, University of Magdeburg, Magdeburg, Germany; 3 Department of Internal Medicine, Rheumatology and Clinical Immunology/Research Center ImmunoSciences (RCIS), Charité - University Medicine Berlin, Berlin, Germany; University at Buffalo, United States of America

## Abstract

**Background:**

Within one week following peroral high dose infection with *Toxoplasma (T.) gondii*, susceptible mice develop non-selflimiting acute ileitis due to an underlying Th1-type immunopathology. The role of the innate immune receptor nucleotide-oligomerization-domain-2 (NOD2) in mediating potential extra-intestinal inflammatory sequelae including the brain, however, has not been investigated so far.

**Methodology/Principal Findings:**

Following peroral infection with 100 cysts of *T. gondii* strain ME49, NOD2^-/-^ mice displayed more severe ileitis and higher small intestinal parasitic loads as compared to wildtype (WT) mice. However, systemic (i.e. splenic) levels of pro-inflammatory cytokines such as TNF-α and IFN-γ were lower in NOD2^-/-^ mice versus WT controls at day 7 p.i. Given that the immunopathological outcome might be influenced by the intestinal microbiota composition, which is shaped by NOD2, we performed a quantitative survey of main intestinal bacterial groups by 16S rRNA analysis. Interestingly, Bifidobacteria were virtually absent in NOD2^-/-^ but not WT mice, whereas differences in remaining bacterial species were rather subtle. Interestingly, more distinct intestinal inflammation was accompanied by higher bacterial translocation rates to extra-intestinal tissue sites such as liver, spleen, and kidneys in *T. gondii* infected NOD2^-/-^ mice. Strikingly, intracerebral inflammatory foci could be observed as early as seven days following *T. gondii* infection irrespective of the genotype of animals, whereas NOD2^-/-^ mice exhibited higher intracerebral parasitic loads, higher F4/80 positive macrophage and microglia numbers as well as higher IFN-γ mRNA expression levels as compared to WT control animals.

**Conclusion/Significance:**

NOD2 signaling is involved in protection of mice from *T. gondii* induced acute ileitis. The parasite-induced Th1-type immunopathology at intestinal as well as extra-intestinal sites including the brain is modulated in a NOD2-dependent manner.

## Introduction

The obligate intracellular parasite *Toxoplasma (T.) gondii* belongs to the Apicomplexa phylum and is widely used in distinct murine models of infection [1]. Low dose (i.e. between 1 and 20 cysts) infection models mimic the course of human *T. gondii* infection such as chronic *Toxoplasma* infection as well as *Toxoplasma* encephalitis [1], [2], [3], [4], [5]. In addition, molecular mechanisms underlying resistance of mice against oral *T. gondii* infection and small intestinal inflammation, for instance, are commonly studied in high dose infection with 50 to 100 cysts of a type II strain [1], [6], [7]. Within one week following peroral infection with 100 cysts of the *T. gondii* ME49 strain, susceptible animals such as C57BL/6 mice (with H-2^b^ haplotype) develop a pan-ileitis and succumb to the infection [1], [2], [8]. Acute ileitis is caused by a Th1-type hyper-inflammatory response characterized by a CD4+ T-cell mediated increase of pro-inflammatory cytokines such as nitric oxide (NO), TNF-α and IFN-γ, whereas *T. gondii* induced counter-regulatory mediators include IL-10 [8], [9]. Hence, the high dose infection model mimics some key features of human inflammatory bowel diseases (IBD) such as Crohn's disease in the acute stage [1], [10], [11]. Furthermore, the ileal immunopathology is associated with an overgrowth of the intestinal microbiota with commensal Gram-negative bacterial species such as *E. coli* which aggravate inflammation via toll-like receptor (TLR) -4 mediated sensing of lipopolysaccharide [1], [11], [12].

The nucleotide-binding oligomerization domain (NOD)-like receptors act as intracellular pattern recognition receptors and regulate host immunity by sensing microbial products and damage-associated signals [13]. Among these, NOD2 encoded by the *card15* gene is expressed in dendritic cells [14], macrophages [15], Paneth cells [16], epithelial cells [17] and at low levels also in T cells [18]. NOD2 confers resistance against a broad variety of bacteria and is activated by muramyl dipeptide (MDP) derived from virtually all Gram-positive as well as Gram-negative bacterial species [13], [19], [20], [21]. However, whether NOD2 senses other microbes and structures or participates only as signaling partner is currently under debate [22]. To date, controversy exists whether NOD2 plays a role in the defense against *T. gondii* infection *in vivo* [13], [23], [24]. In one study, NOD2^-/-^ mice were unable to clear the parasite due to an insufficient Th1-dependent IFN-γ production [24], whereas in another report NOD2^-/-^ mice did not exhibit an enhanced susceptibitily to *T. gondii* infection [23]. Conflicting results might be due to differences in the intestinal microbiota composition of mice used in the respective studies, for instance [22], [23]. The role of NOD2 in mediating potential extra-intestinal inflammatory sequelae including the central nervous system (CNS), however, has not been investigated so far. In the present study we applied the oral high dose *T. gondii* infection model in order to investigate the impact of NOD2 in intestinal, systemic and extra-intestinal immune responses and performed a comprehensive quantitative molecular survey of the intestinal microbiota composition of NOD2^-/-^ and wildtype (WT) mice before and 7 days after *T. gondii* infection.

## Results

### *T. gondii* induced ileal immunopathology is aggravated in NOD2^-/-^ mice

**Figure 1:**
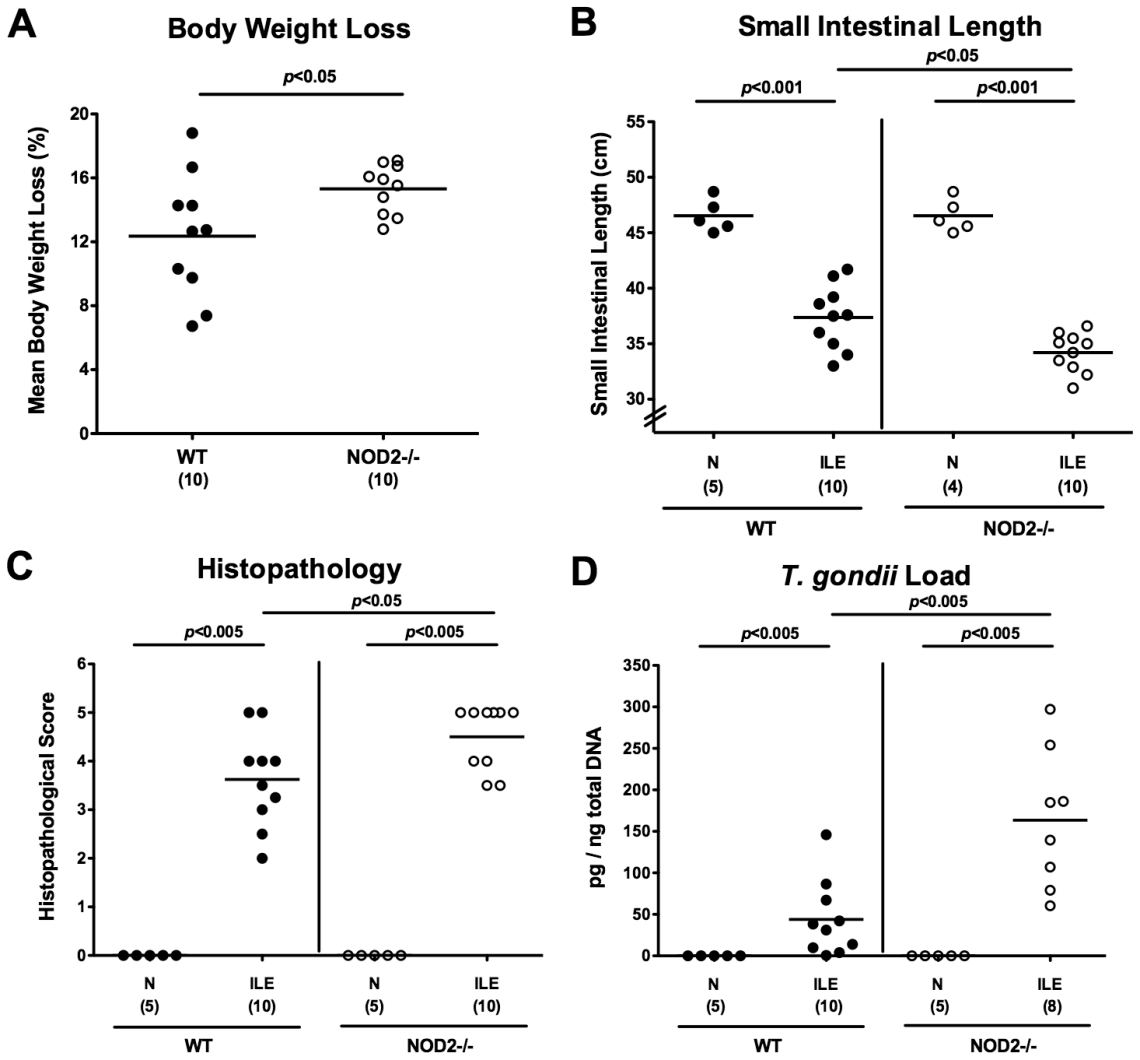
Acute ileitis in *T. gondii* infected NOD2 deficient mice. In order to induce acute ileitis, C57BL/6 wildtype (WT; black circles) and NOD2 deficient (NOD2-/-; white circles) were perorally infected with *T. gondii* at day 0. (**A**) Relative body weight loss between day 7 post infection (p.i.) and day 0 were determined (in %). (**B**) Absolute small intestinal lengths were measured in naïve (N) and *T. gondii* infected mice with ileitis (ILE). (**C**) Histopathological mucosal changes were assessed in ileal paraffin sections, and (**D**) *T. gondii* DNA determined in *ex vivo* ileal biopsies. Numbers of analyzed mice are given in parentheses. Means (black bars) and significance levels (*p*-values) determined by Mann-Whitney-U test are indicated. Data shown are representative for three independent experiments.

In order to investigate the role of NOD2 in *T. gondii* infection and induced acute ileitis, NOD2^-/-^ and WT control mice were perorally infected with 100 cysts of *T. gondii* ME49 strain by gavage at day 0. Until day 7 post infection (d7 p.i.), NOD2^-/-^ animals had lost significantly more body weight as compared to WT mice (15.3±1.5% vs 12.4±4.1%; p<0.05; **Fig. 1A**), indicative of a more compromised clinical condition of the former. Given that acute intestinal inflammation is accompanied by significant shortening of the intestine [11], [25], [26], we measured the individual small intestinal lengths at time of necropsy. At day 7 p.i., NOD2^-/-^ mice displayed significantly shorter small intestines as compared to WT controls (p<0.05; **Fig. 1B**), pointing towards a more distinct small intestinal pathology upon peroral *T. gondii* challenge. We next assessed the degree of histopathological changes in ileal paraffin section. At day 7 p.i., NOD2^-/-^ mice exhibited more inflamed ileal mucosa including necroses as indicated by higher histopathological scores as compared to WT mice (4.5±0.6 versus 3.6±0.9; p<0.05; **Fig. 1C** **and Fig. S1**). Remarkably, NOD2^-/-^ mice harbored approximately three times more *T. gondii* DNA in their ilea as compared to WT mice (p<0.005; **Fig. 1D**).

**Figure 2:**
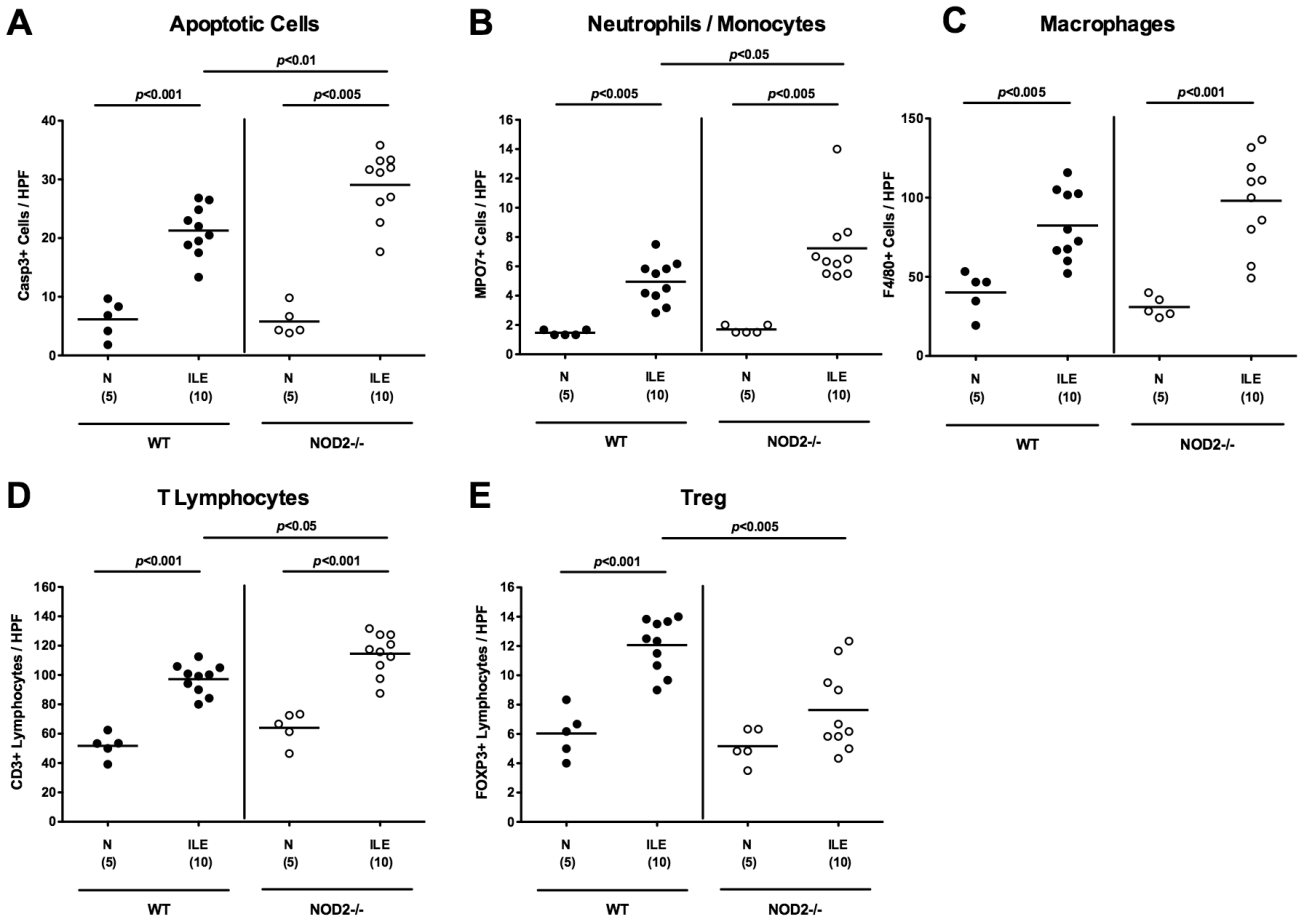
Intestinal immune cell responses following ileitis induction in NOD2 deficient mice. Immune cell responses were assessed microscopically in ileal paraffin sections derived from C57BL/6 wildtype (WT; black circles) and NOD2 deficient (NOD2-/-; white circles) seven days following ileitis induction (ILE) applying *in situ* immunohistochemistry. The average numbers of ileal (**A**) apoptotic cells (positive for caspase-3), (**B**) neutrophilic granulocytes and monocytes (positive for MPO-7), (**C**) macrophages (positive for F4/80), (**D**) T-lymphocytes (positive for CD3), and (**E**) regulatory T-cells (Treg, positive for FOXP3) were determined microscopically in six high power fields (HPF, 400× magnification) per animal. Naive mice served as negative controls (N). Numbers of analyzed mice (in parentheses), means (black bars) and levels of significance (*P*-values) as compared to the respective groups (determined by Mann-Whitney-U test) are indicated. Data shown are representative for three independent experiments.

To further characterize *T. gondii* induced inflammatory responses in the small intestinal tract we next quantitatively determined apoptotic cells as well as distinct immune cell populations in the ileal mucosa and lamina propria by *in situ* immunohistochemical stainings of small intestinal paraffin sections. The ilea of NOD2^-/-^ mice contained approximately 50% more apoptotic cells as compared to WT mice (p<0.01; **Fig. 2A** **and Fig. S2**), further supporting the more devastating clinical and histopathological outcome upon *T. gondii* infection. In addition, a more distinct influx of MPO7 positive cells such as neutrophils and monocytes exerting oxidative stress to the ileal mucosa and lamina propria of NOD2^-/-^ mice could be detected at d7 p.i. (p<0.05; **Fig. 2B** **and Fig. S3**), whereas numbers of F4/80 positive macrophages increased comparably upon *T. gondii* infection in mice of either genotype (p<0.005–0.001 vs naïve mice; **Fig. 2C** **and Fig. S4**). Given that *T. gondii* induced ileitis is a mainly T cell driven pro-inflammatory scenario, we next stained ileal paraffin sections for CD3. The marked increase of T cells induced by *T. gondii* was even more pronounced in NOD2^-/-^ versus WT animals at day 7 p.i. (p<0.05; **Fig. 2D** **and Fig. S5**). Whereas in WT mice a significant, approximately two-fold influx of FOXP3+ regulatory T cells (Treg) into the small intestinal mucosa could be observed at day 7 p.i. (p<0.001 vs naïve control mice; **Fig. 2E** **and Fig. S6**), Treg numbers were significantly lower in NOD2^-/-^ mice following ileitis induction (p<0.05; **Fig. 2E** **and Fig. S6**) and did not differ compared to uninfected, naïve NOD2^-/-^ animals.

**Figure 3:**
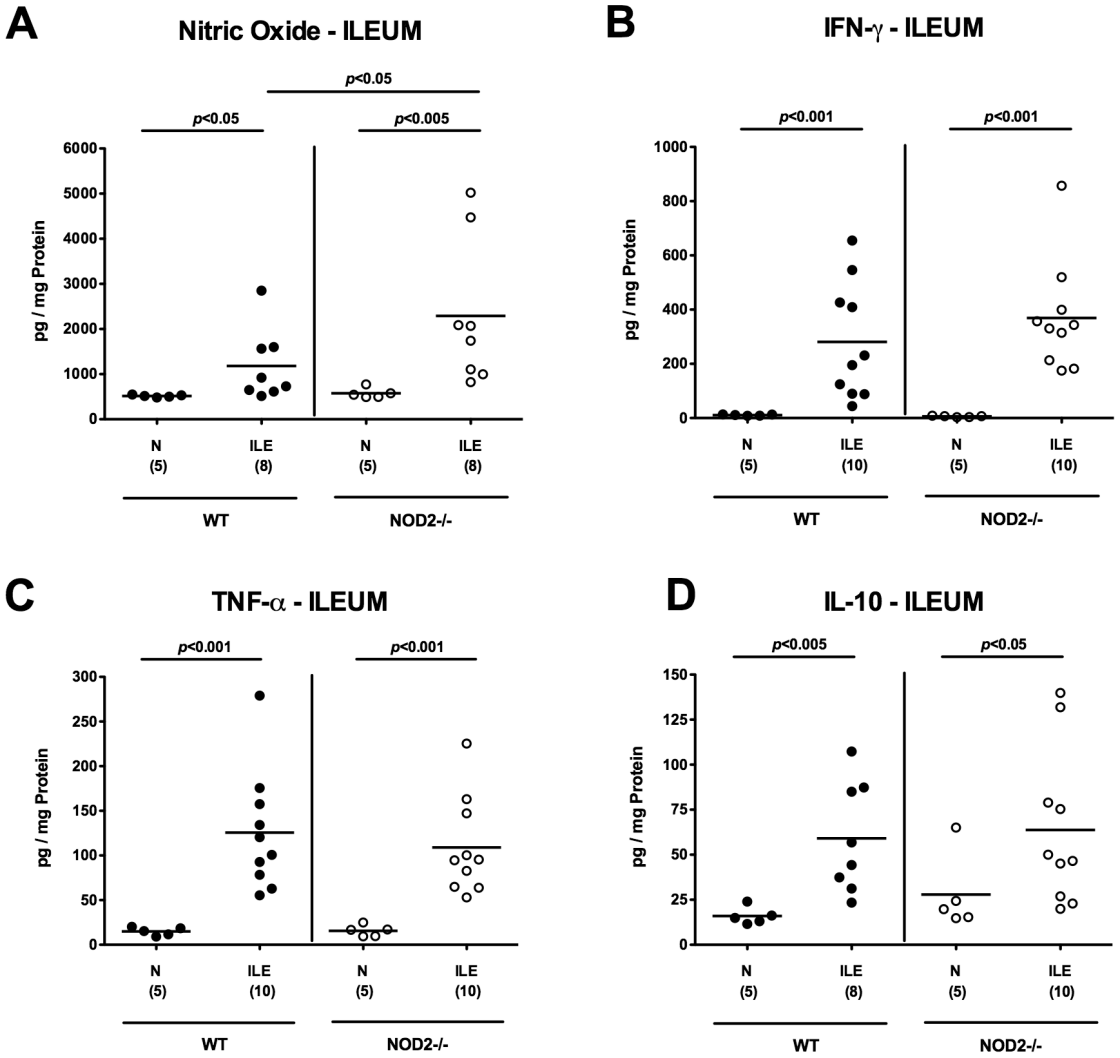
Small intestinal cytokine responses in NOD2 deficient mice following ileitis induction. (**A**) Nitric oxide, (**B**) IFN-γ, (**C**) TNF-α, and (**D**) IL-10 levels were determined in *ex vivo* ileal biopsies derived from C57BL/6 wildtype (WT; black circles) and NOD2 deficient (NOD2-/-; white circles) seven days following ileitis induction (ILE) as described in methods. Naive mice served as negative controls (N). Numbers of analyzed mice (in parentheses), means (black bars) and levels of significance (*P*-values) as compared to the respective groups (determined by Mann-Whitney-U test) are indicated. Data shown are representative for three independent experiments.

We next determined local cytokine secretion in *ex vivo* biopsies derived from ilea of infected and uninfected mice. Irrespective of the genotype of mice, pro-inflammatory mediators such as nitric oxide (NO), IFN-γ and TNF-α increased multi-fold upon ileitis induction (p<0.05–0.001 vs respective naïve controls; **Fig. 3A–C**). Seven days following *T. gondii* infection, higher NO (p<0.05; **Fig. 3A**), but similar IFN-γ and TNF-α protein levels (**Fig. 3B, C**) could be detected in ilea taken from diseased NOD2^-/-^ as compared to WT mice, whereas expression levels of the anti-inflammatory cytokine IL-10 were also multi-fold increased upon *T. gondii* infection, but comparably high in WT and NOD2^-/-^ mice at day 7 (**Fig. 3D**). Taken together, the elevated ileal parasite loads and aggravated inflammation in NOD2^-/-^ mice indicates that NOD2 mediated sensing is essential for protection of mice against *T. gondii* infection.

### NOD2^-/-^ mice display less distinct systemic pro-inflammatory immune responses following *T. gondii* infection

**Figure 4:**
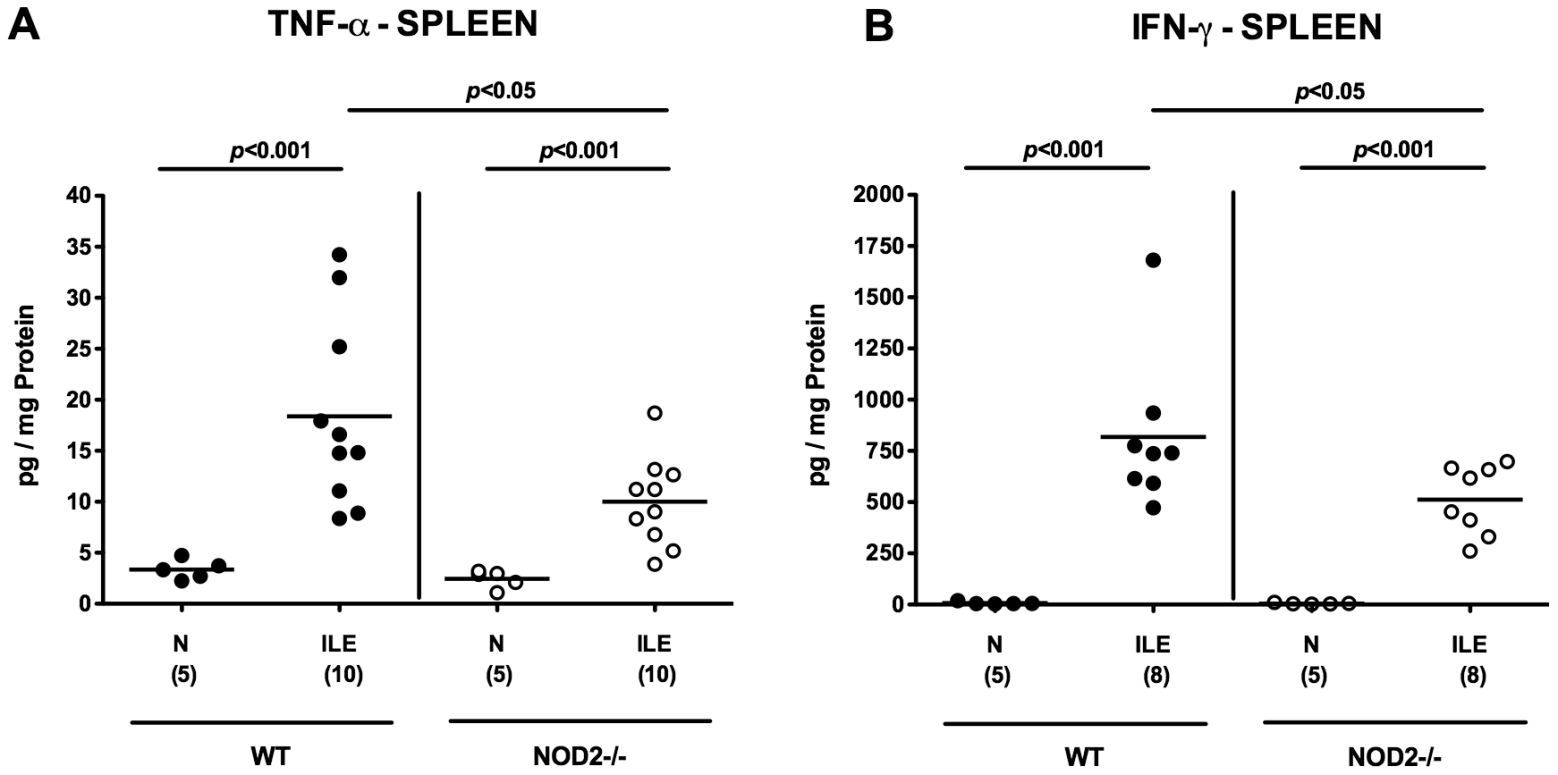
Pro-inflammatory cytokine responses in spleens of NOD2 deficient mice following ileitis induction. Systemic pro-inflammatory cytokine responses were assessed by measuring (**A**) TNF-α and (**B**) IFN-γ levels in *ex vivo* biopsies of spleens derived from C57BL/6 wildtype (WT; black circles) and NOD2 deficient (NOD2-/-; white circles) mice seven days following ileitis induction (ILE). Naive mice served as negative controls (N). Numbers of analyzed mice (in parentheses), means (black bars) and levels of significance (*P*-values) as compared to the respective groups (determined by Mann-Whitney-U test) are indicated. Data shown are representative for three independent experiments.

Next, we assessed potential systemic immune responses upon *T. gondii* infection. To address this, we determined pro-inflammatory cytokine secretion in *ex vivo* biopsies of spleens derived from NOD2^-/-^ and WT mice suffering from acute ileitis. Seven days following *T. gondii* infection, secretion of TNF-α and IFN-γ had increased multi-fold in spleens of either genotype (p<0.001; **Fig. 4**). Remarkably, NOD2^-/-^ mice displayed significantly lower splenic TNF-α and IFN-γ levels as compared to WT mice at day 7 p.i. (p<0.05; **Fig. 4**). Hence, upon ileitis induction NOD2^-/-^ mice displayed more distinct local pro-inflammatory cytokine concentrations (i.e. in inflamed ilea) as compared to WT controls, whereas systemic (i.e. splenic) pro-inflammatory immune responses were less pronounced at day 7 p.i.

### Intestinal microbiota changes in NOD2^-/-^ mice during *T. gondii* ileitis

**Figure 5:**
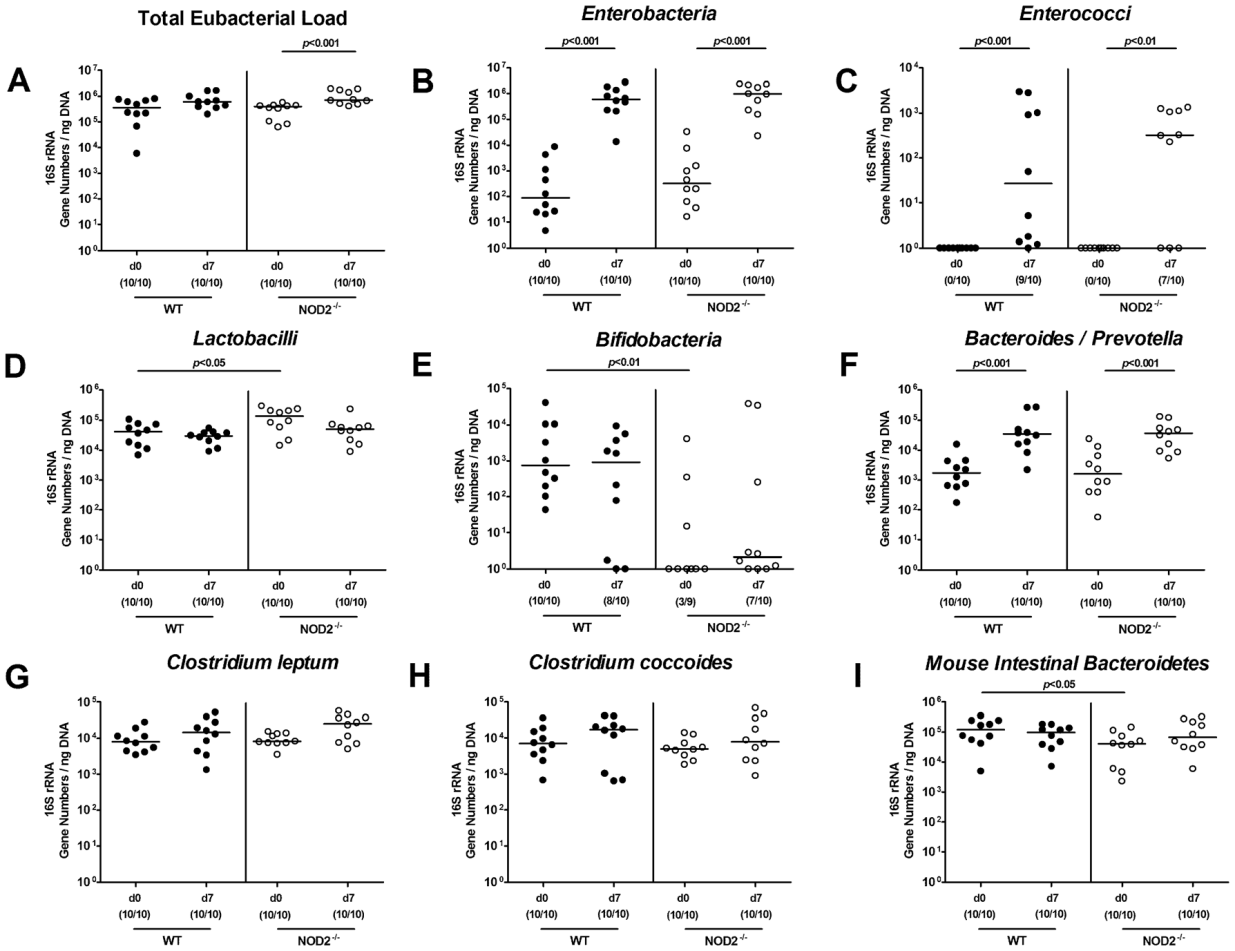
Intestinal microbiota composition of NOD2 deficient mice following ileitis induction. Main bacterial groups of the commensal intestinal microbiota were quantified by molecular analysis of fecal samples derived from C57BL/6 wildtype (WT; black circles) and NOD2 deficient (NOD2-/-; white circles) mice before (N, naïve) and seven days after ileitis induction by peroral *T. gondii* infection (ILE). Quantitative Real-Time-PCR analyses amplified bacterial 16S rRNA variable regions and 16S rRNA gene numbers/ng DNA from the following bacterial groups: (**A**) Total eubacterial load, (**B**) *Enterobacteria*, (**C**) *Enterococci*, (**D**) *Lactobacilli*, (**E**) *Bifidobacteria*, (**F**) *Bacteroides/Prevotella* spp., (**G**) *Clostridium leptum* group, (**H**) *Clostridium coccoides* group, and (**I**) *Mouse intestinal Bacteroidetes*. Numbers of mice harboring the respective bacterial 16S rRNA out of the total number of analyzed animals are given in parentheses. Medians and significance levels (*p*-values) determined by Mann-Whitney-U test are indicated. Data shown are representative for three independent experiments.

Given that the commensal intestinal microbiota composition is a major determinant of susceptibility for and resistance against bacterial or parasitic infection and thus for the inflammatory outcome in the host [27], we performed a comprehensive quantitative molecular survey of the main bacterial groups within the commensal intestinal microbiota during *T. gondii* infection. Overall, in naïve healthy mice differences between age and sex matched NOD2^-/-^ and WT mice were rather subtle (**Fig. 5**). However, NOD2^-/-^ mice harbored slightly higher Lactobacilli, but lower Bifidobacteria, which were virtually absent, and Mouse Intestinal *Bacteriodetes* group in fecal samples (**Fig. 5D, E, I**) as compared to WT mice. In acute ileitis, fecal loads of Enterobacteria, Enterococci, and *Bacteroides/Prevotella* spp. increased in WT as well as NOD2^-/-^ mice (**Fig. 5B, C, F**), whereas only in NOD2^-/-^ mice the total eubacterial load slightly increased until day 7 p.i. (**Fig. 5A**).

### Role of NOD2 in translocation of viable intestinal bacteria to extra-intestinal compartments during *T. gondii* ileitis

**Figure 6:**
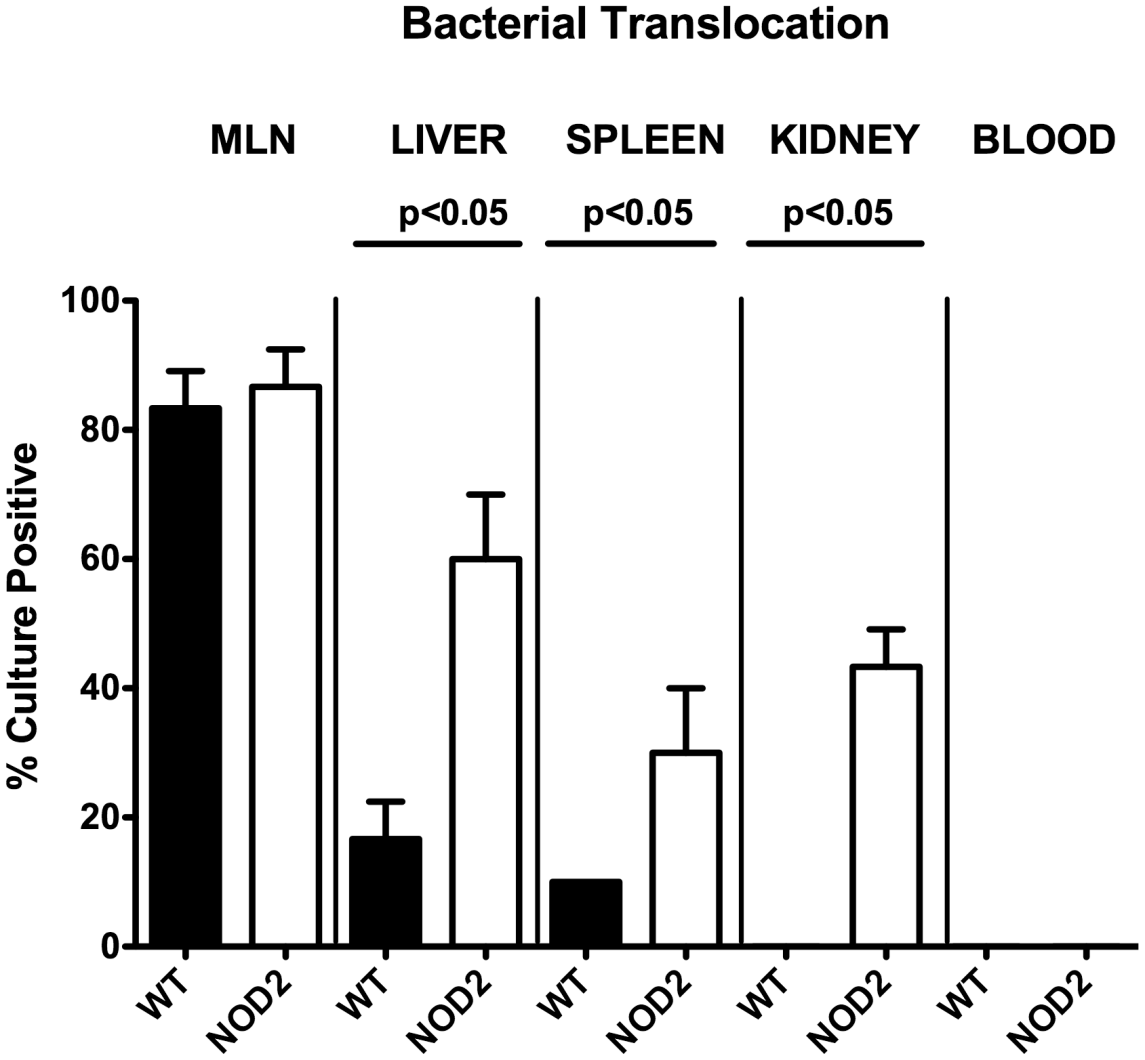
Bacterial translocation in NOD2 deficient following ileitis induction. Relative translocation frequencies (%) of live bacteria were determined in *ex vivo* biopsies of mesenteric lymphnodes (MLN), liver, spleen, kidneys, and blood derived from C57BL/6 wildtype (WT; black bars; n = 10) and NOD2^-/-^ (NOD2; white bars; n = 10) mice by culture in enrichment broths seven days after ileitis induction. Medians, standard deviations and significance levels (*p*-values) determined by Mann-Whitney-U test are indicated. Data shown are pooled from three independent experiments.

We next investigated whether more distinct ileal inflammation and subsequently more compromised epithelial barrier function was in turn accompanied with higher rates of bacterial translocation to extra-intestinal tissue sites. Bacterial translocation rates in liver (60.0±8.17% vs 16.7±4.71%, respectively; p<0.05), spleen (30.0±8.17% vs 10.0±0%, respectively; p<0.05), and kidneys (30.0±8.17% vs 0.0±0.00%, respectively; p<0.05) were higher in NOD2^-/-^ as compared WT mice at day 7 p.i., but comparable in draining mesenteric lymphnodes (MLN: 83.3±4.71% vs 86.7±4.71%, respectively; n.s.) (**Fig. 6**). Translocated viable bacteria comprised aerobic species such as *E. coli*, *Enterococcus* spp., and *Lactobacillus* spp. derived from the commensal intestinal microbiota as determined by culture (not shown). Of note, all cardiac blood samples remained culture-negative (**Fig. 6**). Furthermore, no bacterial translocation at all could be detected in healthy naïve mice (not shown).

### More distinct inflammatory sequelae in brains of NOD2^-/-^ mice following ileitis induction

**Figure 7:**
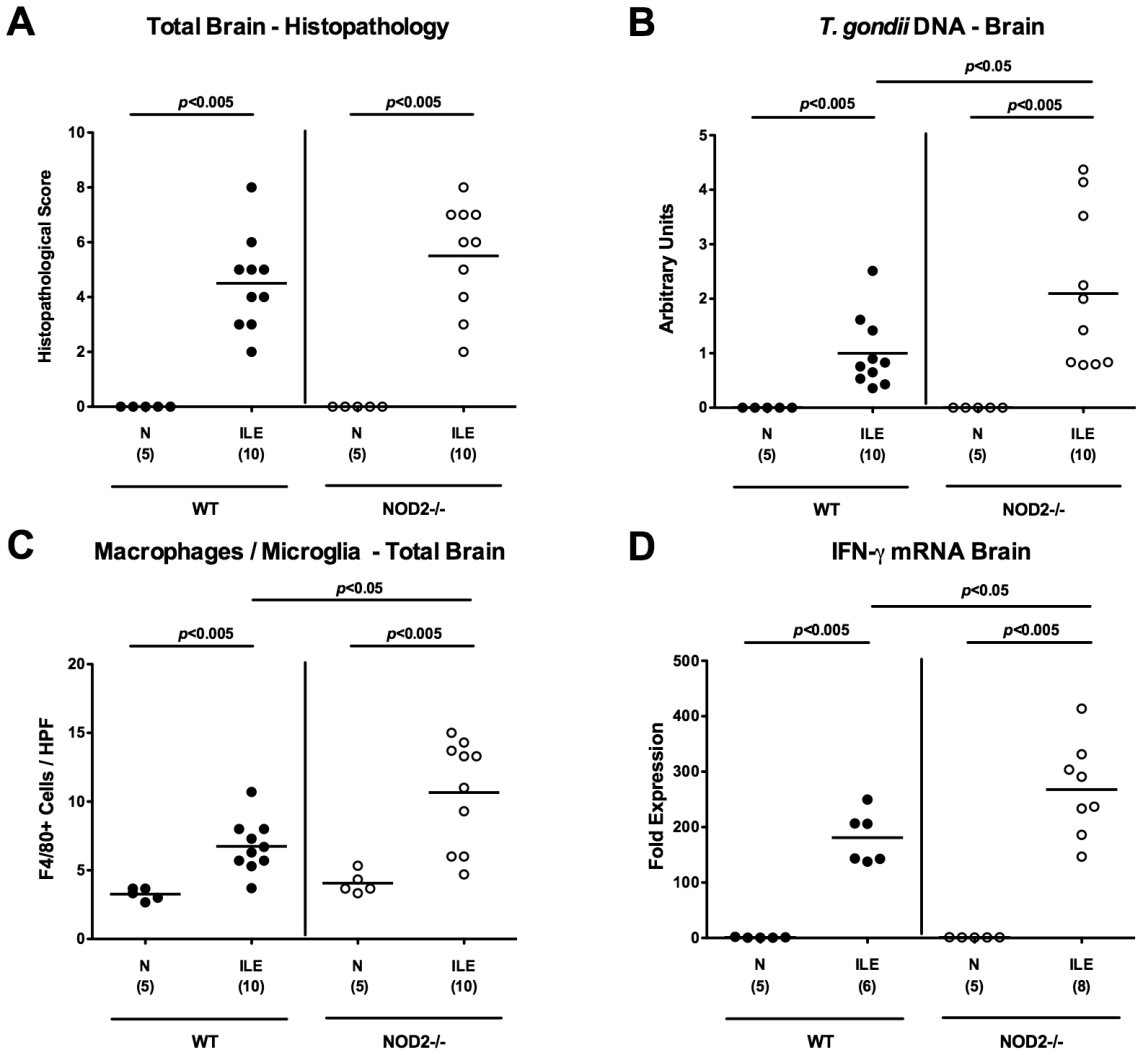
Intracerebral immunopathology in NOD2 deficient mice following ileitis induction. (**A**) Intracerebral histopathological changes (in cortex and meninges) were assessed in H&E stained brain paraffin sections applying a standardized score and (**B**) *T. gondii* DNA determined in *ex vivo* whole brain biopsies derived from C57BL/6 wildtype (WT; black circles) and NOD2 deficient (NOD2-/-; white circles) mice seven days following ileitis induction (ILE) by semi-quantitative RT-PCR and normalized relative to the *M. musculus ASL* gene (Arbitrary Units). Naive mice served as negative controls (N). Furthermore, (**C**) average numbers of macrophages and microglia (positive for F4/80) were quantified microscopically in six high power fields (HPF, 400× magnification) of brain paraffin sections per animal. (**D**) IFN-γ mRNA expression levels were determined in *ex vivo* whole brain biopsies by quantitative RT-PCR. Numbers of analyzed mice (in parentheses), means (black bars) and levels of significance (*P*-values) as compared to the respective groups (determined by Mann-Whitney-U test) are indicated. Data shown are representative for three independent experiments.

To date no data about immunopathological intracerebral changes in the acute ileitis model exist, given that mice succumb the high dose *T. gondii* infection before significant changes in the CNS might become detectable. Remarkably, mice of either genotype displayed significant but comparable intracerebral histopathological changes of both, meninges and cortex as early as 7 days p.i., (p<0.005 vs naïve controls; **Fig. 7A** **and Fig. S7**), whereas the intracerebral *T. gondii* loads measured by RT-PCR were significantly higher in NOD2^-/-^ as compared to WT mice (p<0.05; **Fig. 7B**). Furthermore, brains of NOD2^-/-^ mice exhibited higher numbers of F4/80+ recruited macrophages and microglia cells as compared to WT mice 7 days upon ileitis induction (p<0.05; **Fig. 7C** **and Fig. S8**). The increased numbers of pro-inflammatory immune cells was accompanied by higher expression levels of IFN-γ mRNA in brains of *T. gondii* infected NOD2^-/-^ as compared to WT control mice (p<0.05; **Fig. 7D**). Hence, *T. gondii* induced small intestinal inflammation was accompanied by translocation of intestinal bacteria, systemic dissemination of parasites, and pro-inflammatory responses in the brain that were more pronounced in NOD2^-/-^ as compared to WT mice.

## Discussion

It is well established that NOD2 plays an important role in the innate host defense against intestinal bacterial pathogens such as *Salmonella enteritidis*, *Listeria monocytogenes*, and *Streptococcus pneumoniae* [17], [28], [29], [30]. Results of the present study indicate that NOD2 signaling is also essential for the protection of mice against intestinal parasite infection followed by systemic inflammation including the brain. This was demonstrated by an aggravated clinical condition, more detrimental intestinal inflammation, increased systemic parasite dissemination and brain inflammation in NOD2^-/-^ mice as compared to WT controls. Increased concentrations of parasitic DNA in the intestines and brains of NOD2^-/-^ mice indicated that the aggravation of intestinal and cerebral inflammation was due to enhanced susceptibility to parasitic infection. Enhanced parasitic infection and dissemination in NOD2^-/-^ mice was accompanied by higher translocation rates of viable intestinal bacteria to extra-intestinal compartments such as liver, spleen, and kidney. Our data are partly supported by a previous study demonstrating less clearance of *T. gondii* due to a compromised Th1-dependent IFN-γ production (measured in sera) of infected NOD2^-/-^ mice [24]. In our study, however, local (i.e. ileal) protein expression levels of IFN-γ and TNF-α, which is also involved in parasitic host defense, did not differ between mice of either genotype, but were lower in spleens of infected NOD2^-/-^ as compared to WT mice. Shaw and colleagues further reported that WT infected mice survived the infection, whereas NOD2^-/-^ had all died until day 21 p.i., but data regarding intestinal histopathology were lacking. In our study, however, *T. gondii* challenged mice were severely suffering from acute ileitis, succumbed to the infection and presented in a prefinal condition seven days following infection. In another study and in inconsistency with the report by Shaw and colleagues, Caetano et al. demonstrated that NOD2^-/-^ mice were fully capable of inducing Th1 immune responses and did not display enhanced susceptibility to *T. gondii* infection [23], [24]. Many factors might be responsible for the divergent results in murine infection models. For instance, strain differences and numbers of *T. gondii* cysts as well as the application route might impact the outcome of infection experiments. In both studies by Shaw et al. and Caetano et al., 25 cysts of the ME49 strain were applied intraperitoneally [23], whereas we here challenged our mice with a high dose (i.e. 100 cysts) of the same *T. gondii* strain, although perorally. In addition, Benson et al. demonstrated that NOD2^-/-^ mice exhibited a normal Th1 immunity against *T. gondii* ME49 following high dose peroral infection [31]. One needs to take into consideration that differences in sex, age, diet and the genetic background of the applied mice (incomplete versus complete backcrossings) might have resulted in the observed differences. Notably, a plethora of recent studies highlights the impact of the commensal microbiota composition on initiating, mediating, and perpetuating acute and chronic immunopathology in mice and men [32], [33], [34], [35]. Marked differences in the commensal intestinal microbiota composition might represent a general complication when comparing *in vivo* studies from different research institutions. It is well known that the colonization status of mice ultimately varies between animal facilities, units within the same facility, between rooms within the same unit and additionally between cages within the same room [36], [37], [38]. Furthermore, NOD2 has been shown to be required for orchestrating the commensal intestinal microbiota composition in mice and men [27], [39], and differences are considered to impact the early immune responses following murine infection with intestinal pathogens such as *Citrobacter rodentium* and *Salmonella enterica* Typhimurium [22], [40]. Our quantitative molecular survey of main intestinal bacterial groups revealed that under identical housing conditions in our mouse facility, the overall intestinal microbiota composition differed slightly between naïve NOD2^-/-^ and WT control mice. Remarkably, the Bifidobacteria population was virtually absent in NOD2^-/-^ mice. Eventhough these differences might be rather subtle at the first glance, the biological impact might be significant in our parasitic infection model given that particularly Bifidobacteria are considered as beneficial, probiotic bacterial species with anti-inflammatory properties and, hence, contribute to host colonization resistance against invading pathogens [41]. This is further supported by a previous *in vitro* study, demonstrating that co-culture of Bifidobacteria stimulated dendritic cells with CD4+ T cells resulted in an increase of CD25+ FOXP3+ Tregs [42]. It can be speculated that the observed virtual absence of bifidobacterial species in NOD2^-/-^ mice might at least in part contribute to a compromised host resistance, diminished local anti-inflammatory (i.e. lower intestinal mucosal FOXP3 cell numbers) as well as systemic pro-inflammatory responses (i.e. lower splenic IFN-γ and TNF-α) insufficiently combating peroral *T. gondii* infection.

Intraperitoneal low dose (i.e. max. 20 cysts) *T. gondii* infection of mice represents a model for chronic encephalitis developing within several weeks p.i. [1], [4], [43]. So far, data about intracerebral immunopathological changes following oral high dose *T. gondii* infection are lacking given that acute ileitis develops within one week p.i. and mice succumb before significant changes in the CNS might become detectable. Our results demonstrate for the first time, that intracerebral inflammatory foci could be observed already within seven days following *T. gondii* infection irrespective of the genotype, whereas NOD2^-/-^ mice exhibited higher intracerebral parasitic loads. The elevated numbers of F4/80 positive recruited macrophages and microglial cells in NOD2^-/-^ mice reveal more distinct inflammatory responses compared to WT controls. In addition, the increased IFN-γ mRNA expression in NOD2^-/-^ mice further indicates that NOD2 plays a role not only in the immunopathological processes in the ileum, but also in CNS inflammation.

In conclusion, NOD2 is essentially involved in murine host protection against peroral *T. gondii* infection, parasite-induced Th1-type intestinal immunopathology, parasite dissemination and cerebral inflammation in the murine *T. gondii* induced acute ileitis model.

## Materials and Methods

### Ethics Statement

All animal experiments were conducted according to the European Guidelines for animal welfare (2010/63/EU) and to the ARRIVE guidelines with approval of the commission for animal experiments headed by the “Landesamt für Gesundheit und Soziales” (LaGeSo, Berlin; registration number G0146/10). Animal welfare was monitored twice daily by assessment of clinical conditions and weight loss of mice. Mice suffering from weight loss >20% were euthanized by isofluran treatment (Abbott, Germany) in accordance with the guidelines of the local commission for animal experiments headed by the “Landesamt für Gesundheit und Soziales”. Hence, humane endpoints were implemented in our animal research protocol.

### Mice and induction of acute ileitis

NOD2^-/-^ mice (in C57BL/6 background) were purchased from Jackson Laboratories [28] and were bred and housed together with WT controls under specific pathogen-free (SPF) conditions in the Forschungseinrichtung für Experimentelle Medizin (FEM, Charité – University Medicine Berlin, Germany). For induction of acute ileitis, age matched 3 months old female mice were infected perorally by gavage with 100 *T. gondii* cysts (ME49 strain) from homogenized brains of intraperitoneally infected NMRI mice in a volume of 0.3 mL phosphate-buffered saline (PBS), as described previously [11], [44], [45], [46]. Animals suffering from severe ileitis and succumbing to infection were humanely euthanized.

### Sampling procedures and histopathology

Mice were sacrificed by isofluran treatment (Abbott, Germany). Tissue samples from brain, spleen, liver, kidneys, mesenteric lymphnodes (MLNs) and ileum were removed under sterile conditions. Small intestinal samples from each mouse were collected in parallel for histopathological, immunohistochemical, microbiological, and immunological analyses. Small intestinal lengths were determined by measuring the distance from the duodenum leaving the stomach to the ileal-caecal transiltion by a ruler and expressed in cm. Immunohistopathological changes were determined in samples derived from ileum and brain that were immediately fixed in 5% formalin and embedded in paraffin. Sections (5 µm) were stained with hematoxylin and eosin (H&E), examined by light microscopy (magnification 100× and 400×) and histopathological changes quantitatively assessed applying respective histopathological scoring systems for blinded duplicate evaluation.

**Ileal histopathology** was determined as described previously (according to [11]; max 6 points: 0, normal; 1, minimal focal inflammation, edematous blubbing, intact epithelium; 2, mild inflammation of mucosa and submucosa, cell-free exudate into the lumen, but intact epithelium; 3, moderate inflammation of mucosa and submucosa, erosions and/or ulcerations, cryptitis or crypt abscesses, cellular shedding into the lumen; 4, severe inflammation of mucosa and submucosa, ulcerations, fibrosis, distortion of villous architecture, beginning epithelial disintegration; 5, severe inflammation, mucosal destruction <50% of small intestine length; 6, severe inflammation, complete destruction >50% of small intestine length, severe necroses.

**Brain histopathology** (max. 10 points; according to [2], [4], [43] with minor modifications): Cortex and meningi (separately): number of inflammatory foci per HPF (100× magnification) were assessed. 0, heathy brain structure with no inflammatory foci; 1, single inflammatory foci (1–3); 2, inflammatory foci (4–6); 3, inflammatory foci (7–10); 4, inflammatory foci (11–15); 5, inflammatory foci (>15).

### Immunohistochemistry

*In situ* immunohistochemical analyses of 5 µm thin sections of ileum and whole brain tissue samples that were immediately fixed in 5% formalin and embedded in paraffin before were performed as described previously [26], [47], [48], [49], [50]. Primary antibodies against cleaved caspase-3 (Asp175, Cell Signaling, USA, 1∶200), CD3 (M20, Santa Cruz, 1∶1000), myeloperoxidase-7 (MPO-7, # A0398, Dako, 1∶10000), FOXP3 (FJK-16s, eBioscience, 1∶100) and F4/80 (# 14-4801, clone BM8, eBioscience, 1∶50) were used. For each animal, the average number of positively stained cells within at least six high power fields (HPF, 0.287 mm^2^; 400× magnification) was determined microscopically by two independent double-blinded investigators.

### Quantification of ileal *T. gondii* DNA and measurement of cytokines

Ileal *ex vivo* biopsies were cut longitudinally and washed in PBS. The content of *T. gondii* was quantified by PCR analysis of DNA isolated from approximately 1 cm^2^ of homogenized ileal tissue as described previously [51]. Furthermore, spleen or strips of approximately 1 cm^2^ ileal tissue were placed in 24-flat-bottom well culture plates (Nunc, Wiesbaden, Germany) containing 500 µL serum-free RPMI 1640 medium supplemented with penicillin (100 U/mL) and streptomycin (100 µg/mL; PAA Laboratories). After 18 h incubation at 37°C, culture supernatants were analyzed for IFN-γ, TNF-α, and IL-10 by the Mouse Inflammation Cytometric Bead Assay (CBA; BD Biosciences) in a BD FACSCanto II flow cytometer (BD Biosciences). Nitric oxide (NO) was determined by Griess reaction as described earlier [11].

### Molecular analysis of the intestinal microbiota

Total DNA from fecal samples was extracted as described previously [11]. Briefly, DNA was quantified using Quant-iT PicoGreen reagent (Invitrogen, UK) and adjusted to 1 ng per µL. Then, main bacterial groups abundant in the murine conventional intestinal microbiota were detected by quantitative real-time (RT) -PCR with primers specific for sequences in the 16S rRNA genes of individual bacterial species, genera or groups (Tib MolBiol, Germany) as described previously [47], [48], [52]. Numbers of 16S rRNA gene copies/ng DNA of each sample were determined and frequencies of respective bacterial groups calculated proportionally to the eubacterial (V3) amplicon.

### Bacterial translocation

For qualitative detection of bacterial translocation, MLNs, liver, spleen, kidney and cardiac blood (0.5 mL) were transferred into a thioglycolate broth each and incubated for maximum seven days at 37°C [53]. Bacterial growth was monitored daily by turbidity assessment. Aliquots of turbid broths were cultivated on respective solid media under aerobic, microaerophilic and obligate anaerobic conditions. Bacterial species identification was performed as described earlier [11].

### Cerebral cytokine and parasitic DNA detection

Brain tissue preparation and measurement of IFN-γ mRNA expression by quantitative RT-PCR were performed as described previously [4]. Perfused brain tissue samples were snap-frozen and kept at −80°C. 30 mg of brain tissue were used for nucleic acid purification using the spin column based AllPrep DNA/RNA/Protein Mini Kit (QIAgen, Hilden, Germany) following the manufacturer's instructions. On-membrane DNase I digestion (peqGOLD, Erlangen, Germany) was performed during RNA purification. RNA and DNA purity and concentration were determined by absorbance at 230, 260 and 280 nm in a NanoDrop spectrophotometer (Fisher Scientific, Germany).

Semi-quantitative real time PCR analyses were performed to determine parasite loads in brains. FastStart Essential DNA Green Master (Roche, Grenzach-Wyhlen, Germany) was used with 90 ng genomic DNA in a reaction volume of 20 µL. Triplicate reactions were developed in a LightCycler 480 Instrument II (Roche, Grenzach-Wyhlen, Germany). After an initial activation step (95°C for 10 min), 45 amplification cycles were run, comprising of denaturation at 95°C for 15 sec, annealing at 60°C for 15 sec and elongation at 72°C for 15 sec. The following primers manufactured by Tib MolBiol (Berlin, Germany) were used at a final concentration of 0.3 µM: *Toxoplasma gondii* B1: (Forward) 5′- TCCCCTCTgCTggCgAAAAgT-3′ and (Reverse) 5′-AgCgTTCgTggTCAACTATCgATTg-3′ [54]. *Mus musculus* argininosuccinate lyase (ASL) gene: (Forward) 5′-TCTTCgTTAgCTggCAACTCACCT-3′ and (Reverse) 5′-ATgACCCAgCAgCTAAgCAgATCA-3′ [55].

Parasite loads (target: *Toxoplasma gondii*, B1 gene) were measured relative to mouse cell number (reference: ASL gene), that is the target/reference ratio calculated with the LightCycler 480 Software release 1.5.0 (Roche, Grenzach-Wyhlen, Germany).

To determine relative gene expression, SuperScript III Platinum One-Step Quantitative RT-PCR System (life technologies, Darmstadt, Germany) was used with 300 ng total RNA in a reaction volume of 10 µL. Triplicate reactions were developed in a LightCycler 480 Instrument II (Roche, Grenzach-Wyhlen, Germany). Reverse transcription was performed for 15 min at 50°C followed by 2 min at 95°C. Subsequently, 45 amplification cycles were run, comprising of denaturation at 95°C for 15 sec and annealing/elongation at 60°C for 30 sec. TaqMan Gene Expression Assays (life technologies, Darmstadt, Germany) were used for amplification of *HPRT* (Mm01545399_m1) and *IFNG* (Mm00801778_m1). *HPRT* expression was chosen as reference for normalization and target/reference ratios were calculated with the LightCycler 480 Software release 1.5.0 (Roche, Grenzach-Wyhlen, Germany). Resulting data were further normalized on values of control groups.

### Statistical analysis

Medians, means, standard deviations and levels of significance as determined by Mann-Whitney U-Test were assessed using GraphPad Prism version 6 (GraphPad Software, Inc., San Diego, CA, USA). Two-sided probability (p) values ≤0.05 were considered significant. All experiments were repeated twice.

## Supporting Information

Figure S1 **Histopathological changes in** ***T. gondii*** **infected NOD2 deficient mice suffering from acute ileitis.** Representative photomicrographs of H&E stained ileal paraffin sections illustrate differences in mucosal histopathology seven days following ileitis induction (ILE) in NOD2-/- as compared to wildtype (WT) mice (100× magnification, scale bar 100 µm). Naïve (N) mice served as negative controls.
(TIFF)

Figure S2 **Small intestinal apoptotic cells following ileitis induction in NOD2 deficient mice.** Representative photomicrographs of ileal paraffin sections stained by immunohistochemistry illustrate abundance of apoptotic cells (positive for caspase-3) in small intestines of NOD2-/- as compared to wildtype (WT) mice seven days following ileitis induction (ILE). Naive (N) animals served as negative controls (400× magnification, scale bar 20 µm).
(TIFF)

Figure S3 **Small intestinal abundance of neutrophils and monocytes following ileitis induction in NOD2 deficient mice.** Representative photomicrographs of ileal paraffin sections stained by immunohistochemistry illustrate abundance of neutrophilic granulocytes and monocytes (positive for MPO-7) in small intestines of NOD2-/- as compared to wildtype (WT) mice seven days following ileitis induction (ILE). Naive (N) animals served as negative controls. Arrows indicate positively stained cells (400× magnification, scale bar 20 µm).
(TIFF)

Figure S4 **Small intestinal abundance of macrophages following ileitis induction in NOD2 deficient mice.** Representative photomicrographs of ileal paraffin sections stained by immunohistochemistry illustrate abundance of macrophages (positive for F4/80) in small intestines of NOD2-/- as compared to wildtype (WT) mice seven days following ileitis induction (ILE). Naive (N) animals served as negative controls (400× magnification, scale bar 20 µm).
(TIFF)

Figure S5 **Small intestinal abundance of T lymphocytes following ileitis induction in NOD2 deficient mice.** Representative photomicrographs of ileal paraffin sections stained by immunohistochemistry illustrate abundance of T lymphocytes (positive for CD3) in small intestines of NOD2-/- as compared to wildtype (WT) mice seven days following ileitis induction (ILE). Naive (N) animals served as negative controls (400× magnification, scale bar 20 µm).
(TIFF)

Figure S6 **Small intestinal abundance of regulatory T cells following ileitis induction in NOD2 deficient mice.** Representative photomicrographs of ileal paraffin sections stained by immunohistochemistry illustrate abundance of regulatory T cells (Treg, positive for FOXP3) in small intestines of NOD2-/- as compared to wildtype (WT) mice seven days following ileitis induction (ILE). Naive (N) animals served as negative controls (400× magnification, scale bar 20 µm).
(TIFF)

Figure S7 **Intracerebral immunopathology in NOD2 deficient mice following ileitis induction.** Representative photomicrographs of H&E stained brain paraffin sections illustrate cerebral histopathological changes (meninges and cortex) seven days following ileitis induction (ILE) in NOD2-/- as compared to wildtype (WT) mice. Arrows indicate inflammatory foci (100× magnification, scale bar 100 µm). Naïve (N) mice served as negative controls.
(TIFF)

Figure S8 **Intracerebral macrophages and microglia in NOD2 deficient mice following ileitis induction.** Cerebral macrophages and microglia were visualized following F4/80 staining of brain paraffin sections (400× magnification, scale bar 20 µm) derived seven days following ileitis induction (ILE) in NOD2-/- as compared to wildtype (WT) mice. Naïve (N) mice served as negative controls.
(TIFF)

Checklist S1 **ARRIVE Guidelines Checklist for reporting of** ***in vivo*** **experiments.**
(PDF)
