# Supplementary figures and images for: Nucleotide-Oligomerization-Domain-2 Affects Commensal Gut Microbiota Composition and Intracerebral Immunopathology in Acute Toxoplasma gondii Induced Murine Ileitis
Source: PLoS One. 2014 Aug 20;9(8):e105120. doi: 10.1371/journal.pone.0105120 (PMC4139296; doi:10.1371/journal.pone.0105120)

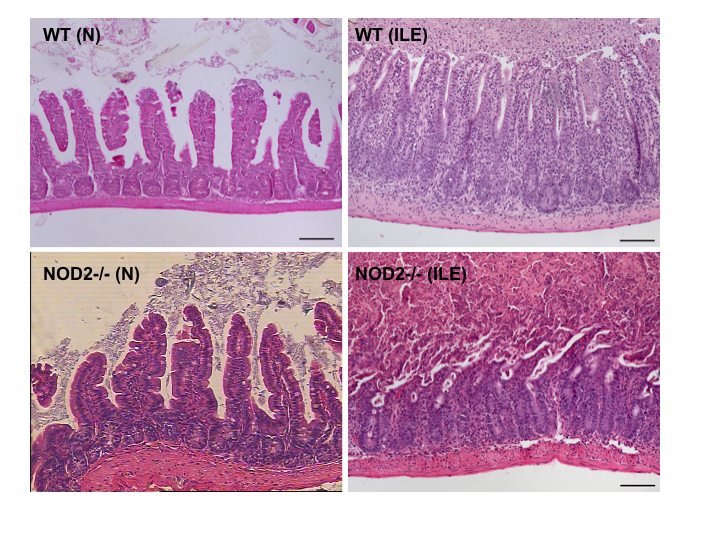

Supplement: Figure S1 — Histopathological changes in T. gondii infected NOD2 deficient mice suffering from acute ileitis. Representative photomicrographs of H&E stained ileal paraffin sections illustrate differences in mucosal histopathology seven days following ileitis induction (ILE) in NOD2-/- as compared to wildtype (WT) mice (100× magnification, scale bar 100 µm). Naïve (N) mice served as negative controls. (TIFF) [file pone.0105120.s001.tiff]

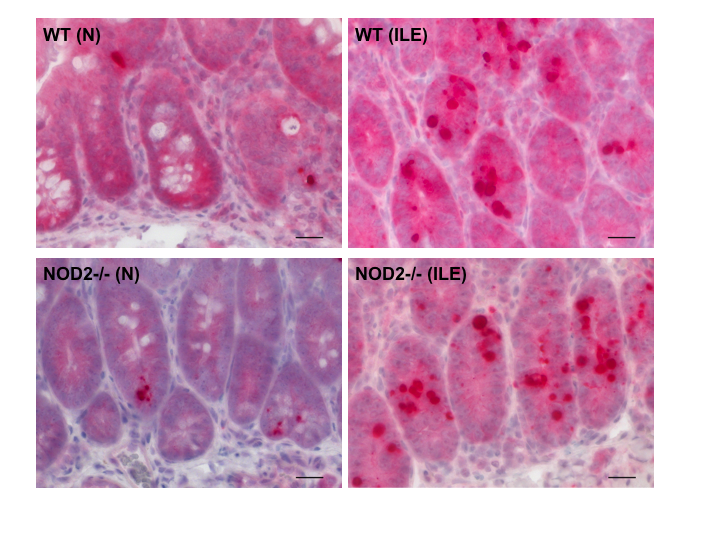

Supplement: Figure S2 — Small intestinal apoptotic cells following ileitis induction in NOD2 deficient mice. Representative photomicrographs of ileal paraffin sections stained by immunohistochemistry illustrate abundance of apoptotic cells (positive for caspase-3) in small intestines of NOD2-/- as compared to wildtype (WT) mice seven days following ileitis induction (ILE). Naive (N) animals served as negative controls (400× magnification, scale bar 20 µm). (TIFF) [file pone.0105120.s002.tiff]

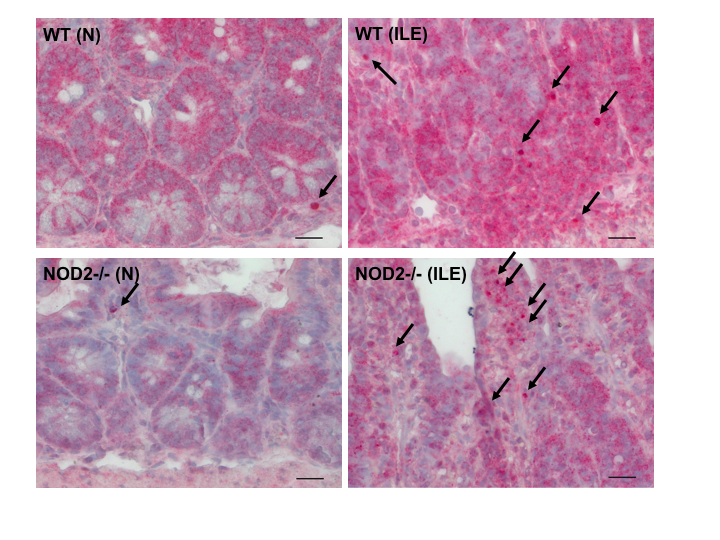

Supplement: Figure S3 — Small intestinal abundance of neutrophils and monocytes following ileitis induction in NOD2 deficient mice. Representative photomicrographs of ileal paraffin sections stained by immunohistochemistry illustrate abundance of neutrophilic granulocytes and monocytes (positive for MPO-7) in small intestines of NOD2-/- as compared to wildtype (WT) mice seven days following ileitis induction (ILE). Naive (N) animals served as negative controls. Arrows indicate positively stained cells (400× magnification, scale bar 20 µm). (TIFF) [file pone.0105120.s003.tiff]

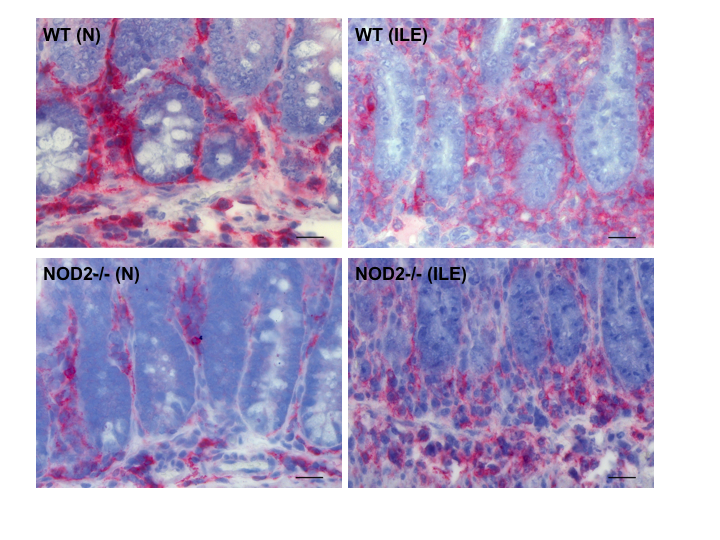

Supplement: Figure S4 — Small intestinal abundance of macrophages following ileitis induction in NOD2 deficient mice. Representative photomicrographs of ileal paraffin sections stained by immunohistochemistry illustrate abundance of macrophages (positive for F4/80) in small intestines of NOD2-/- as compared to wildtype (WT) mice seven days following ileitis induction (ILE). Naive (N) animals served as negative controls (400× magnification, scale bar 20 µm). (TIFF) [file pone.0105120.s004.tiff]

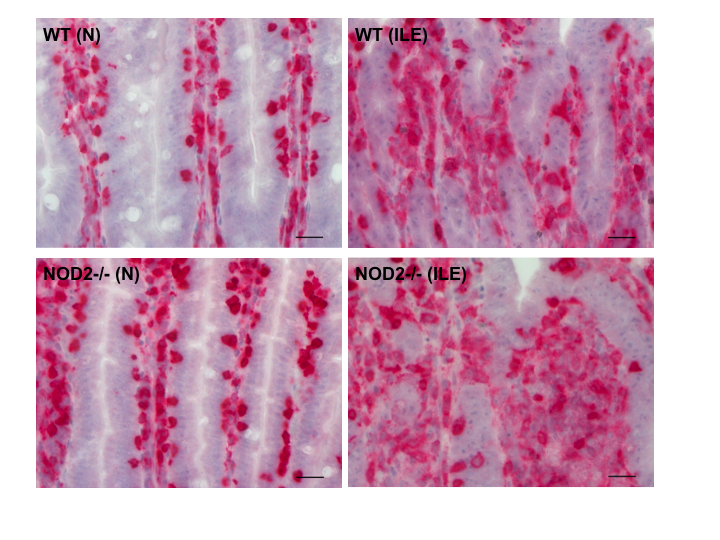

Supplement: Figure S5 — Small intestinal abundance of T lymphocytes following ileitis induction in NOD2 deficient mice. Representative photomicrographs of ileal paraffin sections stained by immunohistochemistry illustrate abundance of T lymphocytes (positive for CD3) in small intestines of NOD2-/- as compared to wildtype (WT) mice seven days following ileitis induction (ILE). Naive (N) animals served as negative controls (400× magnification, scale bar 20 µm). (TIFF) [file pone.0105120.s005.tiff]

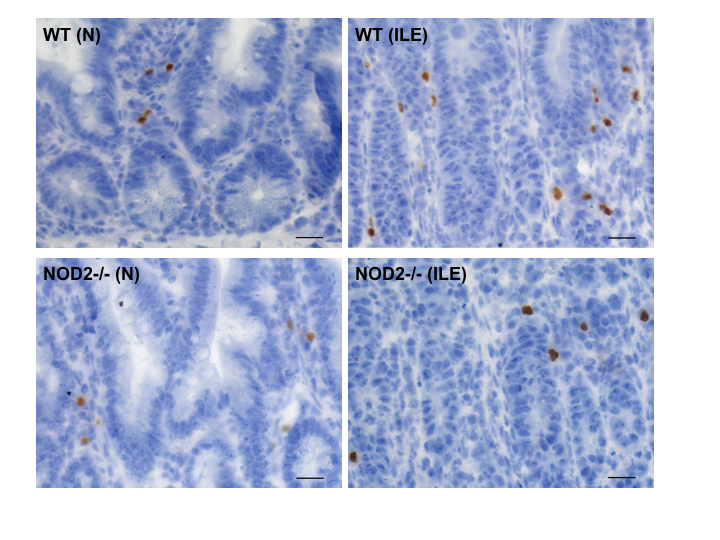

Supplement: Figure S6 — Small intestinal abundance of regulatory T cells following ileitis induction in NOD2 deficient mice. Representative photomicrographs of ileal paraffin sections stained by immunohistochemistry illustrate abundance of regulatory T cells (Treg, positive for FOXP3) in small intestines of NOD2-/- as compared to wildtype (WT) mice seven days following ileitis induction (ILE). Naive (N) animals served as negative controls (400× magnification, scale bar 20 µm). (TIFF) [file pone.0105120.s006.tiff]

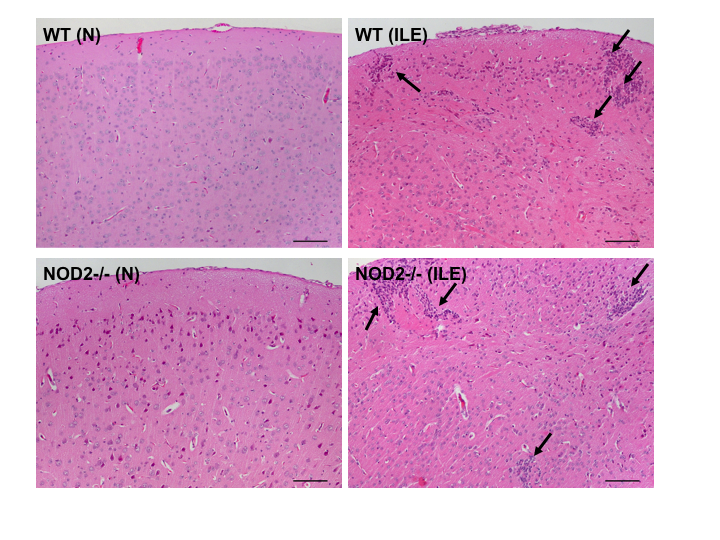

Supplement: Figure S7 — Intracerebral immunopathology in NOD2 deficient mice following ileitis induction. Representative photomicrographs of H&E stained brain paraffin sections illustrate cerebral histopathological changes (meninges and cortex) seven days following ileitis induction (ILE) in NOD2-/- as compared to wildtype (WT) mice. Arrows indicate inflammatory foci (100× magnification, scale bar 100 µm). Naïve (N) mice served as negative controls. (TIFF) [file pone.0105120.s007.tiff]

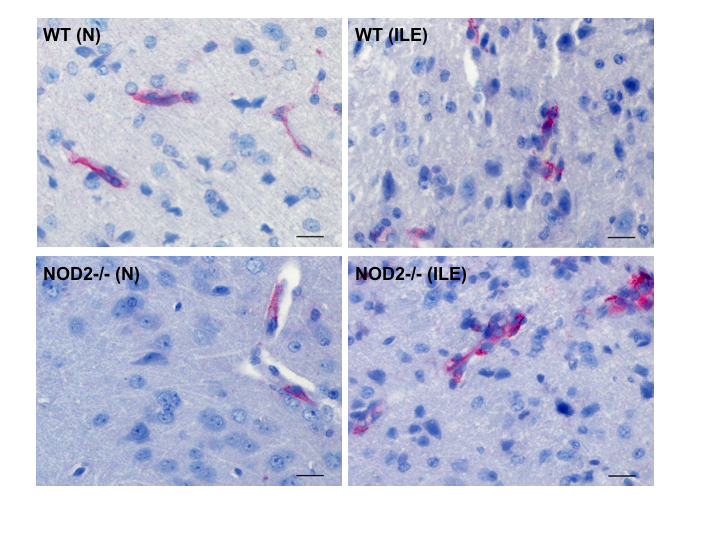

Supplement: Figure S8 — Intracerebral macrophages and microglia in NOD2 deficient mice following ileitis induction. Cerebral macrophages and microglia were visualized following F4/80 staining of brain paraffin sections (400× magnification, scale bar 20 µm) derived seven days following ileitis induction (ILE) in NOD2-/- as compared to wildtype (WT) mice. Naïve (N) mice served as negative controls. (TIFF) [file pone.0105120.s008.tiff]
